# Non-linear optical microscopy and histological analysis of collagen, elastin and lysyl oxidase expression in breast capsular contracture

**DOI:** 10.1186/s40001-018-0322-0

**Published:** 2018-06-04

**Authors:** Patrina S. P. Poh, Verena Schmauss, Jacqui A. McGovern, Daniel Schmauss, Mohit P. Chhaya, Peter Foehr, Markus Seeger, Vasilis Ntziachristos, Dietmar W. Hutmacher, Martijn van Griensven, Jan-Thorsten Schantz, Elizabeth R. Balmayor

**Affiliations:** 1 Experimental Trauma Surgery, Department of Trauma Surgery, Klinikum rechts der Isar, Technical University of Munich, Munich, Germany; 2 Department of Plastic Surgery and Hand Surgery, Klinikum rechts der Isar, Technical University of Munich, Munich, Germany; 3 Institute of Health and Biomedical Innovation, Queensland University of Technology (QUT), Brisbane, Australia; 4 Institute for Advanced Study, Technical University of Munich, Garching, Germany; 5 Department of Orthopaedics and Sports Orthopaedics, Klinikum rechts der Isar, Technical University of Munich, Munich, Germany; 6 School of Chemical and Biomedical Engineering, Nanyang Technological University, Singapore, Singapore; 7 Chair for Biological Imaging, Technical University of Munich, Munich, Germany; 8 Institute of Biological and Medical Imaging, Helmholtz Zentrum München, Neuherberg, Germany

**Keywords:** Silicone breast implant, Collagen, Alpha-smooth muscle actin, Fibrous capsule

## Abstract

**Background:**

Capsular contracture is one of the most common complications in surgical interventions for aesthetic breast augmentation or post-mastectomy breast reconstruction involving the use of silicone prostheses. Although the precise cause of capsular contracture is yet unknown, the leading hypothesis is that it is caused by long-term unresolved foreign body reaction towards the silicone breast implant. To authors’ best knowledge, this is the first study that elucidates the presence of lysyl oxidase (LOX)—an enzyme that is involved in collagen and elastin crosslinking within fibrous capsules harvested from patients with severe capsular contracture. It was hypothesized that over-expression of LOX plays a role in the irreversible crosslinking of collagen and elastin which, in turn, stabilizes the fibrous proteins and contributes to the progression of capsular contracture.

**Methods:**

Eight fibrous capsules were collected from patients undergoing capsulectomy procedure, biomechanical testing was performed for compressive Young’s moduli and evaluated for Type I and II collagen, elastin and LOX by means of non-linear optical microscopy and immunohistology techniques.

**Results:**

Observations revealed the heterogeneity of tissue structure within and among the collected fibrous capsules. Regardless of the tissue structure, it has been shown that LOX expression was intensified at the implant-to-tissue interface.

**Conclusion:**

Our results indicate the involvement of LOX in the initiation of fibrous capsule formation which ultimately contributes towards the progression of capsular contracture.

**Electronic supplementary material:**

The online version of this article (10.1186/s40001-018-0322-0) contains supplementary material, which is available to authorized users.

## Background

Over the last 60 years, silicone breast implants have been widely used in aesthetic and reconstructive breast surgery. The placement of a breast implant initiates a cascade of foreign body responses, starting from an inflammatory reaction followed by recruitment of fibroblasts, which secrete collagen and myofibroblasts, which produce alpha-smooth muscle actin (α-SMA)-positive stress fibers. These events lead to formation of a collagenous capsule around the implant. In the initial stage, the collagenous encapsulation helps to maintain the position of the implant. However, since the foreign body responses persist over time, the collagenous capsule continues to remodel. This leads to excessive fibrous tissue accumulation, which subsequently hardens and tightens. This results in capsular contracture leading to discomfort, pain, deformity and distortion of the breast [1–4]. To date, capsular contracture remains to be the leading cause for patient dissatisfaction of both aesthetic and reconstructive breast implant surgery. The frequency of the clinical manifestation of capsular contracture varies dramatically in patients and may be influenced by a number of exogenous factors, including, but not limited to, surgical technique, trauma, implant fill/surface, incision location, placement relative to pectoralis major muscle, infection, biofilm formation, radiation therapy and others [5]. Clinically, a capsular contracture of the breast is classified by the Baker classification system, whereby Grade 1 is a normal, soft and non-palpable implant; Grade 2 is a slightly firm breast with normal appearance; Grade 3 is a moderately firm breast with abnormal appearance and Grade 4 is a painful and hard breast with distorted implant [6].

It is well established that the matrix compositions of fibrous capsules surrounding a breast implant is predominantly collagen [1, 2, 7, 8]. Through intense research in the field of oncology [9, 10] and dermal wound healing [11, 12], it has been revealed that lysyl oxidase (LOX) is a copper-dependent enzyme, which initiates the process of collagen and elastin cross-linking in the extracellular matrix (ECM) through oxidative deamination of peptidyl lysine and hydroxylysine residues in collagens, and peptidyl lysine residues in elastin. The resulting peptidyl aldehydes spontaneously condense and undergo oxidation reactions to form the lysine-derived covalent cross-links, stabilizing the fibrous deposits of these proteins in the ECM; thus, converting soluble precursor proteins to insoluble fibers [9]. To the best of authors’ knowledge, to date, the presence of LOX has not been elucidated for fibrous capsules surrounding a breast implant. This study hypothesized that LOX plays a role in the irreversible cross-linking of collagen and elastin which stabilizes the fibrous proteins and contributes to the progression of capsular contracture. Hence, in this pilot study, the composition of Type I collagen, Type II collagen, elastin and LOX were investigated in clinical samples harvested from patients suffering from capsular contracture by means of histological analysis and/or non-linear optical (NLO) microscopy.

## Methods

### Fibrous capsule collection

Collection of clinical samples was performed after obtaining permission from the local ethical review committee (ethics approval number: 348/14S) of Klinikum rechts der Isar, Technical University of Munich. Tissue was collected with patient’s informed consent. The study was conducted following the ethical guidelines of the Klinikum rechts der Isar, Technical University of Munich and in accordance with the Declaration of Helsinki in its latest version.

Eight fibrous capsules formed around silicone breast implants with textured surfaces were collected from five patients clinically diagnosed with capsular contracture with Baker score of grade 3 (*N* = 4) and 4 (*N* = 4) (see Table 2). The time of implants residing in the patients ranged from 1.5 to 30 years, as illustrated in Table 1. All patients had undergone aesthetic breast augmentation in the past and were scheduled for revision surgery due to capsular contracture. During the surgery, capsulectomy was performed in all patients and subsequently new implants were inserted. Excised fibrous capsules were collected in phosphate buffered saline (PBS) for biomechanical testing.

**Table 1:** List of samples collected in this study

Sample ID (*N*)	Implant duration (years)	Baker score	Revision surgery	No. of biopsy (*n*)
1	1.5	3	Fourth	8
2	10	3	First	8
3	10	3	First	8
4	12	3	First	8
5	10	4	First	16
6	10	4	First	16
7	30	4	First	16
8	30	4	First	16

### Fibrous capsule compressive Young’s moduli

Biopsies (*Ø* = 8 mm) were obtained from each fibrous capsule (listed in Table 1) and subjected to unconfined compression using a uniaxial test system (Zwicki1120, Zwick and Roell, Ulm, Germany) fitted with a 20 N load cell (class 0.01, A.S.T. Mikrosystemtechnik). All mechanical testing was done within 24 h after sample collection. Since all fibrous capsule biopsies possessed different heights, the height of each specimen was measured to calculate a 30% compression depth applied at a rate of 0.1 mm/s. The tangent at the linear region of the stress–strain graph was used to derive the compressive Young’s modulus.

### Histology and immunohistochemistry

For processing samples into paraffin, tissue biopsies from each fibrous capsule were fixed in 4% paraformaldehyde (PFA) for 24 h. Samples were dehydrated in graded series of ethanol and infiltrated with paraffin using a tissue processor (Excelsior ES, Thermo Scientific) and embedded in paraffin. Tissue sections of 6 µm thickness were taken using a microtome (Leica2265, Germany).

For histological analysis, tissue sections were stained with hematoxylin and eosin (H&E) and Masson Trichrome.

#### Deparaffinization and rehydration

Prior to staining, the tissue sections were deparaffinized (2 changes of Rosti-Histo, 6 min each) and rehydrated in a graded ethanol series (100, 90, and 70% ethanol, 3 min each; dH_2_O for 3 min).

#### H&E

Tissue sections were stained with Mayer’s hematoxylin for 10 min, washed with tap water for 10 min, and stained with Eosin for 2 min.

#### Masson trichrome

Staining was performed using a Masson–Goldner–Trichrome Staining Kit (Carl Roth, Germany) following manufacturer’s instructions. Briefly, tissue sections were stained with Weigert’s iron hematoxylin solution for 2 min and blued in flowing tap water for 10 min. Then, stained with Goldner’s stain I (Poncean-Fuchsin) for 7 min and rinsed with 1% acetic acid for 30 s. Subsequently, stained with Goldner’s stain II (Phosphotungstic acid-Orange G) for 3 min and rinsed with 1% acetic acid for 30 s. Sections were counterstained with Goldner’s stain III (Light green SF yellowish) for 2 min and washed with 1% acetic acid.

#### Dehydration and mounting

Upon completion of the staining procedures, tissue sections were dehydrated through ascending ethanol series (70, 90 and 100% ethanol, 3 min each, and two changes of xylene, 6 min each) and cover-slipped using Eukitt^®^ mounting media (Sigma-Aldrich, Germany).

For immunohistochemistry, samples were deparaffinised and rehydrated. Antigen retrieval was performed using proteinase K (Dako, Australia) treatment at room temperature for 15 min. Subsequently, endogenous peroxidase activity within the sections was quenched by incubating with 3% H_2_O_2_ for 5 min, washed thrice with Dako Wash buffer (2 min each wash), and incubated with Biocare Medical Background SNIPER for 10 min at room temperature to block non-specific binding sites. Then, samples were washed and incubated with primary antibody diluted using Dako EnVision FLEX Antibody diluent (Table 2). To rule out non-specific reactions of rabbit or mouse IgG on the examined sections, non-immunised mouse or rabbit IgG was used as an isotype control. Samples were washed three times in Dako Wash buffer and incubated with peroxidase-labelled dextran polymer-conjugated goat anti-mouse and anti-rabbit immunoglobulins (DAKO Envision + Dual Link System Peroxidase, Australia) at room temperature for 45 min in a humidified chamber. The antibody complexes were visualized by the addition of a buffered diaminobenzidine (DAB)-based system (DAKO). After color development, samples were washed with Dako Wash buffer and counterstained with Mayer’s hematoxylin (HD Scientific Pty. Ltd., Australia) for 2 min and blued in 0.1% ammonium hydroxide for 10 secs. Subsequently, tissue sections were dehydrated in a graded ethanol series and mounted with Eukitt^®^ mounting media (Sigma-Aldrich, Australia). Positive and negative controls were also taken into account (Additional file 1: Figure S1).

**Table 2:** Primary antibody supplier, dilution and incubation time used for immunohistochemistry

Primary antibody	Supplier	Dilution	Incubation time
Collagen 1	Abcam (ab23446)	1:100	Overnight at 4 °C
Alpha-smooth muscle actin	Millipore (CBL171)	1:300	1 h at room temperature
Lysyl Oxidase	Abcam (ab31238)	1:200	Overnight at 4 °C

### Histomorphometry

Histomorphometrical measurements of the fibrous capsule thickness were performed on three consecutive sections of each biopsy (*n* = 8 biopsies per fibrous capsule) using Leica Digital Image Hub web interface. First, a reference line was drawn across the entire tissue section. Subsequently, measurement lines were drawn perpendicular to the reference line (five equally spaced measurement lines per tissue section), defining the fibrotic capsule thickness. The thickness of the fibrotic capsule was derived from the average length of these measurement lines.

### Second and third harmonic generation and two-photon excitation fluorescence microscopy for analysis of elastin, type I collagen and type II collagen

NLO microscopy, namely second and third harmonic generation (SHG and THG), as well as two-photon excitation fluorescence (TPEF), was carried out on a custom-built multiphoton microscope as described previously [13–15]. The system was equipped with a Yb-based solid-state 1043 nm laser (YBIX, Time-Bandwidth, Switzerland), which was guided over a set of high-speed galvanometric mirrors (6215H, Cambridge Technology, USA). The beam was further enlarged by plano-convex lenses in a telescopic arrangement and finally focused by a microscopic objective lens (Plan Apochromat 10X, Zeiss, Jena, Germany; air immersion, NA: 0.45) mounted in an inverted microscope (AxioObserver. D1, Zeiss, Germany) into the sample. For imaging, the beam was raster-scanned across the specimens hold by high-precision xyz-stages (MLS203-2 and MZS500, Thorlabs, USA) in a field of view (FOV) of 638 × 638 µm^2^ with a resolution of 800 × 800 pixels and an image averaging of 100. NLO signals were filtered by corresponding optical filters (SHG (FB520-10), THG (FGUV11), TPEF (FELH0550), Thorlabs), sensed by highly sensitive photomultiplier tubes (H9305-03, Hamamatsu, Japan), and digitized by a 16 bit data acquisition card (PCIe 6363, National Instruments, USA), which was also controlling the scanning of the galvanometric mirrors. THG was detected in forward direction above the sample, whereas SHG and TPEF were detected in backward direction using a short-pass dichroic mirror (DMSP805R, Thorlabs) to separate the signals from the excitation wavelength. The data acquisition, image generation, and control of the microscope was carried out in Labview (National Instruments). Image processing was performed using ImageJ (ImageJ 1.50e, Wayne Rasband, US National Institutes of Health, USA).

## Results

### Compressive Young’s moduli and fibrous capsule thickness

The compressive Young’s modulus and thickness of tested fibrous capsule biopsies (Table 3) revealed high variability within and among the collected fibrous capsules (*n* = 8 or 16 biopsies per fibrous capsule).

**Table 3:** Compressive Young’s modulus (mean ± standard deviation) of individual samples

Sample ID (*N*)	No. of biopsy (*n*)	Baker score	Compressive Young’s modulus (MPa)	Fibrous capsule thickness (µm)
1	8	3	2.59 ± 1.59	1046.3 ± 462.7
2	8	3	3.24 ± 2.12	711.7 ± 177.7
3	8	3	4.1 ± 1.89	825.6 ± 291.3
4	8	3	7.5 ± 5.43	931.1 ± 351.4
5	16	4	1.97 ± 0.88	1705 ± 1115.7
6	16	4	1.57 ± 0.37	1537 ± 752.7
7	16	4	5.69 ± 7.88	1182 ± 353.5
8	16	4	5.36 ± 7.73	782.6 ± 358

### Tissue morphology and composition of fibrous capsules

**Fig. 1:**
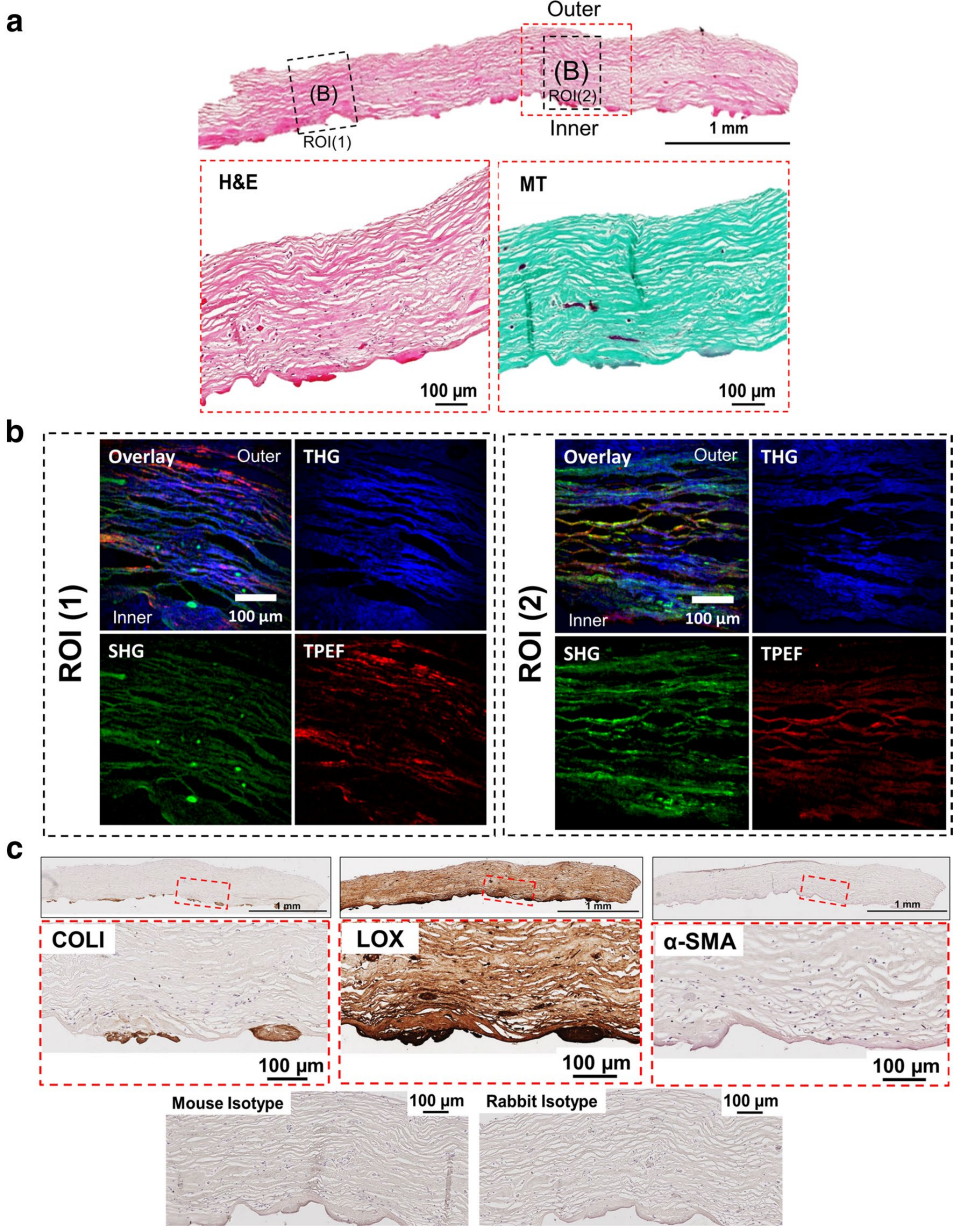
Histological and NLO microscopy images of capsule with Baker grade 3 (example 1). **a** An overview of a capsule biopsy with Baker grade 3 stained with hematoxylin and eosin (H&E) with representative magnified images stained with H&E and Masson Goldner’s Trichrome (MT). *MT* Green stained for connective tissue and red for muscle fibers. **b** Non-linear optical (NLO) microscopy images with second and, third harmonic generation (SHG, THG) and two-photon excitation fluorescence (TPEF) images. *SHG* Collagen I & II (COLI and COLII), *THG* tissue morphology and *TPEF* fibrillar structure represent elastin. **c** Tissues immunohistochemically stained with COLI, lysyl oxidase (LOX) and alpha-smooth muscle actin (α-SMA) with the respective magnified images. Mouse and rabbit isotype were included as negative controls

**Fig. 2:**
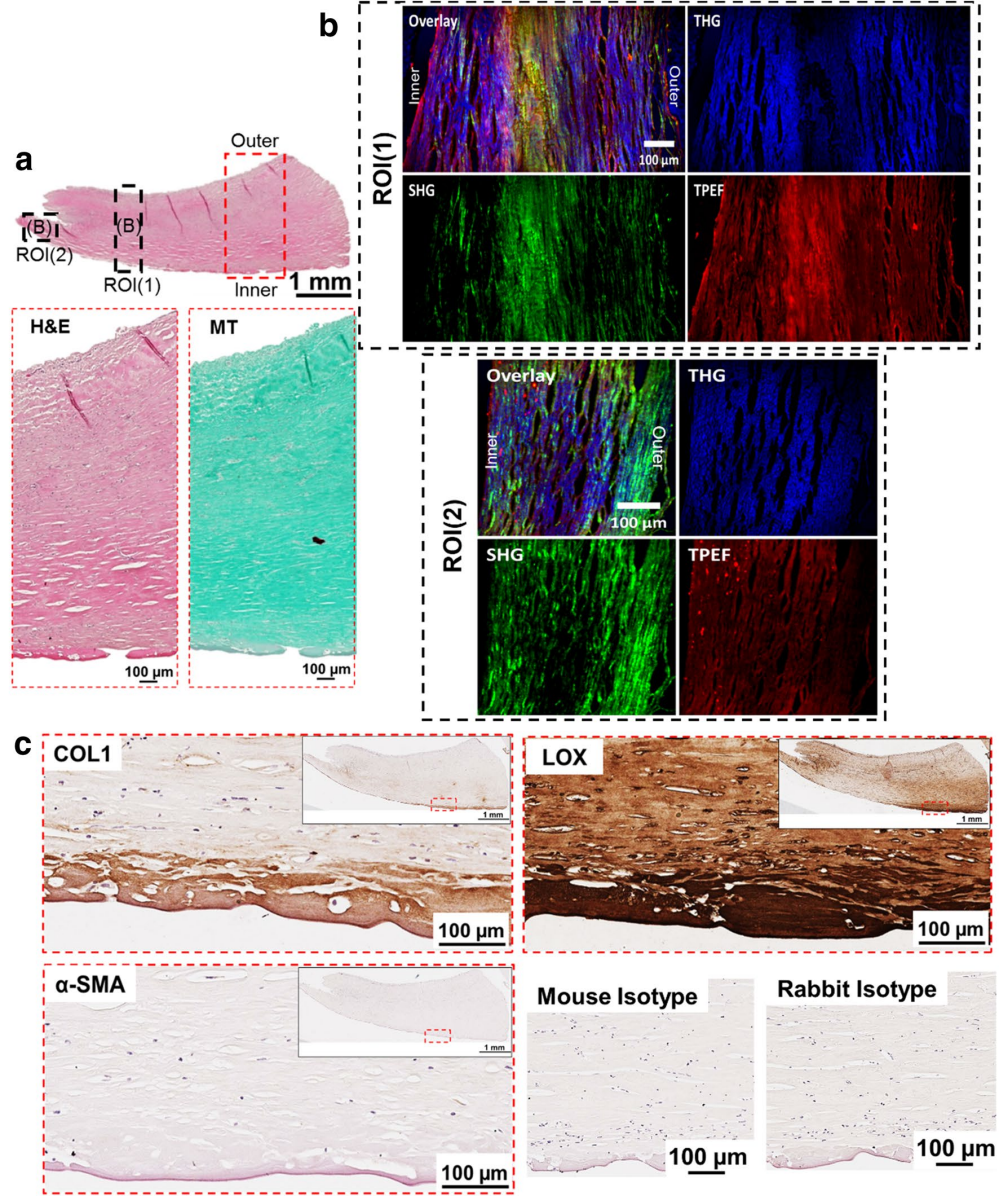
Histological and NLO microscopy images of capsule with Baker grade 3 (example 2). **a** An overview of a capsule biopsy with Baker grade 3 stained with hematoxylin and eosin (H&E) with representative magnified images stained with H&E and Masson Goldner’s Trichrome (MT). *MT* Green stained for connective tissue and red for muscle fibers. **b** Non-linear optical (NLO) microscopy images with secondary- and third harmonic generation (SHG, THG) and two-photon excitation fluorescence (TPEF) images. *SHG* Collagen I & II (COLI and COLII), *THG* tissue morphology and *TPEF* fibrillar structure represent elastin. **c** Tissues immunohistochemically stained with COLI, lysyl oxidase (LOX) and alpha-smooth muscle actin (α-SMA) with the respective magnified images. Mouse and rabbit isotype were included as negative controls

**Fig. 3:**
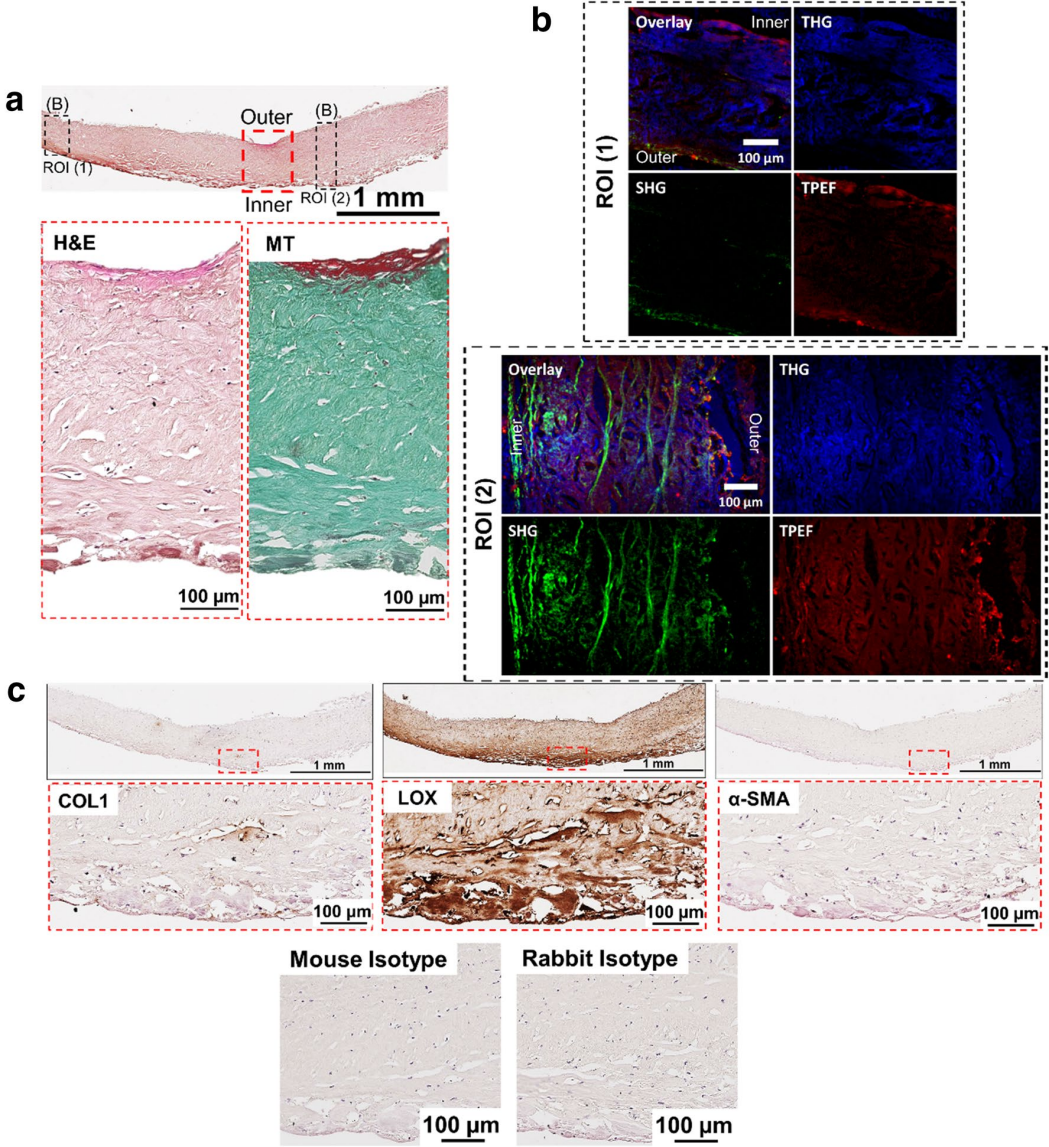
Histological and NLO microscopy images of capsule with Baker grade 4 (example 1). **a** An overview of a capsule biopsy with Baker grade 4 stained with hematoxylin and eosin (H&E) with representative magnified images stained with H&E and Masson Goldner’s Trichrome (MT). *MT* Green stained for connective tissue and red for muscle fibers. **b** Non-linear optical (NLO) microscopy images with secondary-, third harmonic generation (SHG, THG) and two-photon excitation fluorescence (TPEF) images. *SHG* Collagen I & II (COLI and COLII), *THG* tissue morphology and *TPEF* fibrillar structure represent elastin. **c** Tissues immunohistochemically stained with COLI, lysyl oxidase (LOX) and alpha-smooth muscle actin (α-SMA) with the respective magnified images. Mouse and rabbit isotype were included as negative controls

**Fig. 4:**
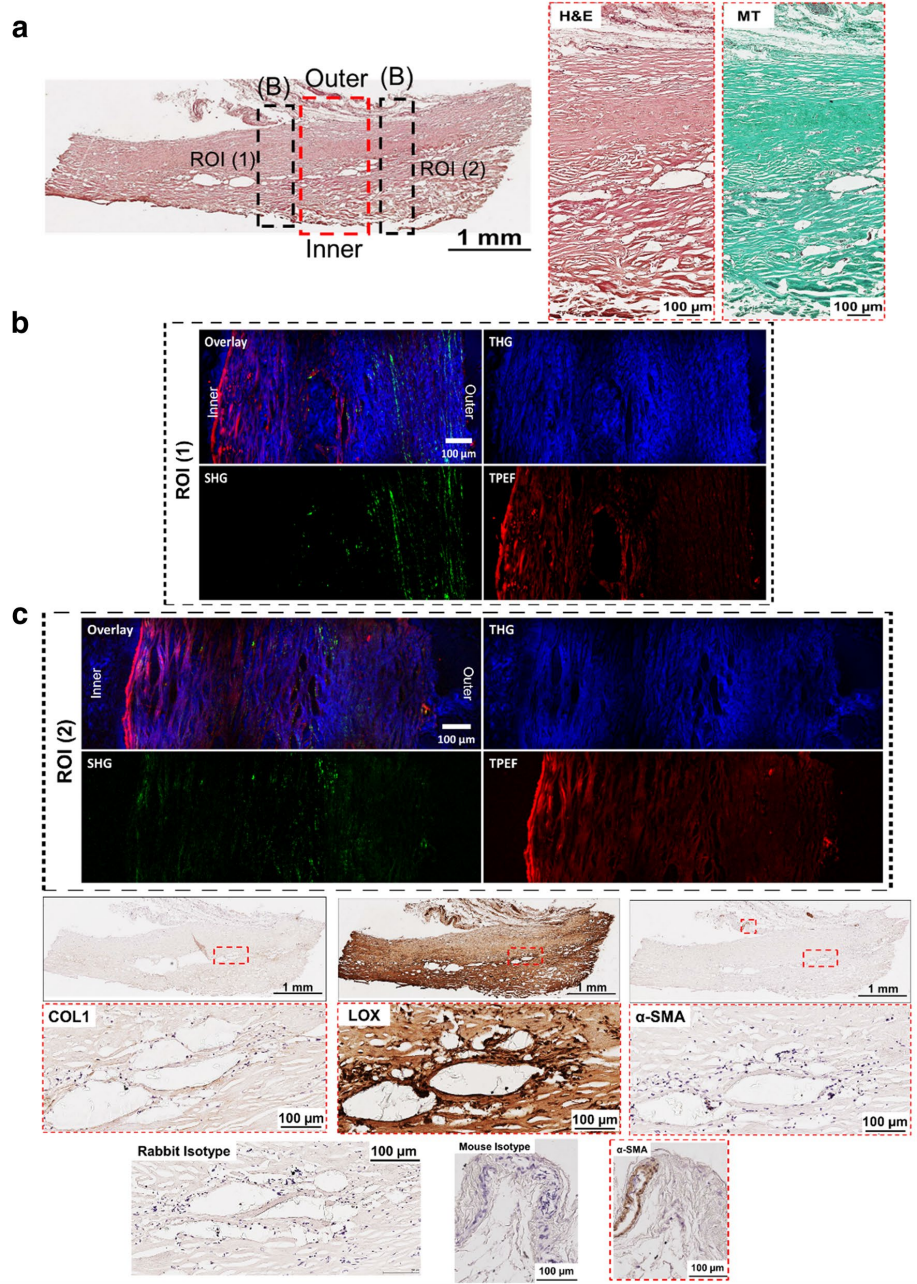
Histological and NLO microscopy images of capsule with Baker grade 4 (example 2). **a** An overview of a capsule biopsy with Baker grade 4 tissues stained with hematoxylin and eosin (H&E) with representative magnified images stained with H&E and Masson Goldner’s Trichrome (MT). *MT* Green stained for connective tissue and red for muscle fibers. **b** Non-linear optical (NLO) microscopy images with secondary-, third-harmonic generation (SHG, THG) and two-photon excitation fluorescence (TPEF) images. *SHG* Collagen I & II (COLI and COLII), *THG* tissue morphology and *TPEF* fibrillar structure represent elastin. **c** Tissues immunohistochemically stained with COLI, lysyl oxidase (LOX) and alpha-smooth muscle actin (α-SMA) with the respective magnified images. Mouse and rabbit isotype were included as negative controls

Regardless of the Baker score, all capsules consist predominantly of collagen fibers with cells sparsely distributed across the entire thickness of the capsule as illustrated in H&E- and MT-stained tissue sections in Figs. 1a, 2a, 3a, 4a. Subsequently, NLO and immunohistological techniques was used to further characterize the composition of the tissue.

#### Fibrous capsule with Baker grade 3

SHG and TPEF microscopy demonstrated that capsules of Baker grade 3 have abundant type I collagen, type II collagen and elastin fibers (Figs. 1b, 2b). It was further verified that type I collagen was highly expressed at the implant-to-tissue interface (Figs. 1c, 2c). Additionally, it was noted that LOX was ubiquitously expressed through the entire capsule biopsy and intensified at the implant-to-tissue interface (Figs. 1c, 2c).

#### Fibrous capsule with Baker grade 4

High heterogeneity was observed for fibrous capsules with Baker grade 4 with the amount of type I and II collagen differs across the tissue length. Figure 3b showed that within the imaged region of interest (ROI) 1, a small amount of type I and II collagen was detected at the tissue distance from the silicone implant (outer layer of the capsule) and a small amount of elastin was detected at the tissue-to-implant interface (inner layer of the capsule). On the other hand, ROI 2 (Fig. 3b) is characterized by an abundance of type I and II collagen, whereas a small amount of elastin is revealed at the outer and inner layers of the sample. In contrast, on Fig. 4b, the type I and II collagen amount was lower compared to that of Baker grade 3 capsules, with notably more type I and II collagen at the outer layer of the capsule. It was observed, that elastin was predominantly present at the inner layer of the capsule. In contrast to fibrous capsule of Baker grade 3, only a small number of localized type I collagen staining was observed within the thickness of the capsules of Baker grade 4 (Figs. 3c, 4c). It was also seen that LOX was ubiquitously present throughout the tissue with higher intensity of LOX expression at the inner layer of the capsule (Figs. 3c, 4c).

Across all groups, no α-SMA was detected (Figs. 1c, 2c, 3c, 4c) within the capsule. In Fig. 4c, blood vessels in tissue distal to the silicone implant was positively stained for α-SMA.

## Discussion

Silicone implants are currently the most popular alloplastic material for breast augmentation and reconstruction. However, they are associated with a substantial risk of developing a capsular contracture. In this study, it was noted that among the tissue biopsies harvested from the same sample, the compressive Young’s modulus and capsule thickness were vastly different (with high standard deviations). This observation correlates with the subsequent histological analyses (i.e., gross tissue morphology, connective tissue-packing density) which show tissue heterogeneity across a capsule sample.

The MT-stained images (Figs. 1a, 2a, 3a, 4a) demonstrated that all capsules consist of predominantly collagen. This observation was aligned with previous histological studies of breast capsules [2, 16, 17]. It is well established that cross-linking of collagen is mediated by LOX through (1) the allysine route, in which lysine (Lys) residue within a collagen telopeptide is converted into aldehyde allysine; or (2) the hydroxylysine route, in which a hydroxylysine (Hyl) residue within a collagen telopeptide is converted into aldehyde hydroxylysine. Subsequently, the allysine or the hydroxylysine reacts with a Lys, Hyl or histidine residue in the triple helix to form di-, tri-, or tetra-functional cross-links [12, 18]. The implications of LOX have been previously studied for several human fibrotic disorders, e.g., kidney fibrosis [19], liver fibrosis [20] and oral submucous fibrosis [21]. However, the implications of LOX on the pathology of capsular contracture have not yet been elucidated. Hence, in this pilot study, the primary aim was to determine whether there is also an over-expression of LOX within capsules with severe contraction (Baker grades 3 and 4) using NLO imaging and immunohistology techniques.

Non-linear optical imaging technique was used in study to enable label-free visualization of selected ECM components of fibrous capsules based on optical harmonic detection or tissue auto-fluorescence as well as their co-localisation on the same tissue section. Studies have shown that SHG microscopy can reveal type I and II collagen structure [22], TPEF microscopy can detect the presence of elastin [23], and THG microscopy can be used for the visualization of samples’ surface morphology [24]. Investigation by means of NLO indicated that capsules of Baker grade 3 have a higher content of type I collagen, type II collagen and elastin compared to capsules of Baker grade 4. Immunohistology revealed that type I collagen is present in capsules of Baker grade 3 at the tissue-implant interface and was almost absent in capsules of Baker grade 4. In contrast, regardless of the fibrous capsules’ baker score, it was observed that LOX was ubiquitously present throughout the collagenous tissue and expression was highest at the tissue-implant interface. Considering the fact that LOX is also involved in elastin cross-linking [25], this two-pronged observation indicates that the collagen composition changes dynamically and that the tissue undergoes high activity of collagen and/or elastin cross-linking as the capsule tissue pathology progresses from Baker 3 to 4, forming cross-linked mature collagen fibrils, which are less susceptible to proteolytic degradation.

Another indication of capsular contracture is the presence of α-SMA-positive myofibroblasts, which play a major role in matrix contraction [26, 27]. It is known that the generation of α-SMA-positive myofibroblasts requires at least three local events in the tissue micro-environment, namely the accumulation of biologically active transforming growth factor-beta 2 (TGF-β2), the presence of ED-A splice variant of fibronectin and high ECM stress [28]. In particular, it has been increasingly accepted that α-SMA is a mechanosensitive protein and only become incorporated into pre-existing β-cytoplasmic actin stress fibers when substrate stiffness permits formation of super-mature focal adhesions (FA) (8–30 μm long) generating approximately fourfold greater stress compared to the usual FA (2–6 μm long) [28, 29]. In this study, no α-SMA-positive myofibroblasts were detected in any samples despite being clinically classified as Baker grade 3 or 4 capsular contractures. Similar findings were reported by Bui et al. [7], whereby α-SMA was not found in capsules harvested from textured implants but ubiquitously expressed in capsules harvested from smooth implants. It is beyond the scope of this study to validate the reason for the absence of α-SMA. However, it is speculated that diminished presence of α-SMA staining in the presence of contracture may highly resemble the process of wound healing and scar formation where myofibroblasts undergo apoptosis in the later stages while tissue contraction persists [7, 30] due to the over-expression of LOX which has been reported to increased tissue stiffness [11, 31].

## Conclusion

In conclusion, it is highly likely that LOX plays a role in the pathology of development of fibrous capsule around silicone breast implant and ultimately contributing to the development of capsular contracture. The presented study has demonstrated that inhibition of LOX activity using lathyrogen β-aminopropionitrile (BAPN) has been reported to reduce fibroblast-mediated contraction on collagen gels [32]. This study indicates that LOX could potentially be used as therapeutic target for the prevention/treatment of capsular contracture. However, further studies are necessary to determine the mode of collagen cross-linking (i.e., allysine or hydroxylysine route) during the progression of capsular contracture. Brinckmann et al. [33] showed that in skin fibrosis, a switch from the allysine to the hydroxylysine route occurs rather than an increase in the total number of enzymatic cross-links, which appears to be an important criterion in assessing the irreversibility of fibrosis [34, 35]. One limitation of this study is the relatively small sample size. Hence, the findings of this study need to be further validated with a larger sample size to ensure that the observed phenomena are ubiquitous across different demographic groups.

## Additional file

**Additional file 1: Figure S1.** Positive/negative control for IHC of Collagen I (COLI), alpha-smooth muscle actin (α-SMA) and Lysyl Oxidase (LOX).

## Abbreviations

LOX: lysyl oxidase
α-SMA: alpha-smooth muscle actin
ECM: extracellular matrix
NLO: non-linear optical
PBS: phosphate buffered saline
PFA: paraformaldehyde
H&E: hematoxylin and eosin
DAB: diaminobenzidine
SHG: second harmonic generation
THG: third harmonic generation
TPEF: two-photon excitation fluorescence
MT: Masson trichrome
TGF-β2: transforming growth factor-beta 2
FA: focal adhesion
BAPN: lathyrogen beta-aminopropionitrile
