# Supplementary material for: Non-linear optical microscopy and histological analysis of collagen, elastin and lysyl oxidase expression in breast capsular contracture
Source: Eur J Med Res. 2018 Jun 4;23:30. doi: 10.1186/s40001-018-0322-0 (PMC5987584; doi:10.1186/s40001-018-0322-0)

**Additional Figure**

**Additional Figure S1:** Positive/Negative control for IHC of Collagen I (COLI), alpha-smooth muscle actin (α-SMA) and Lysyl Oxidase (LOX).


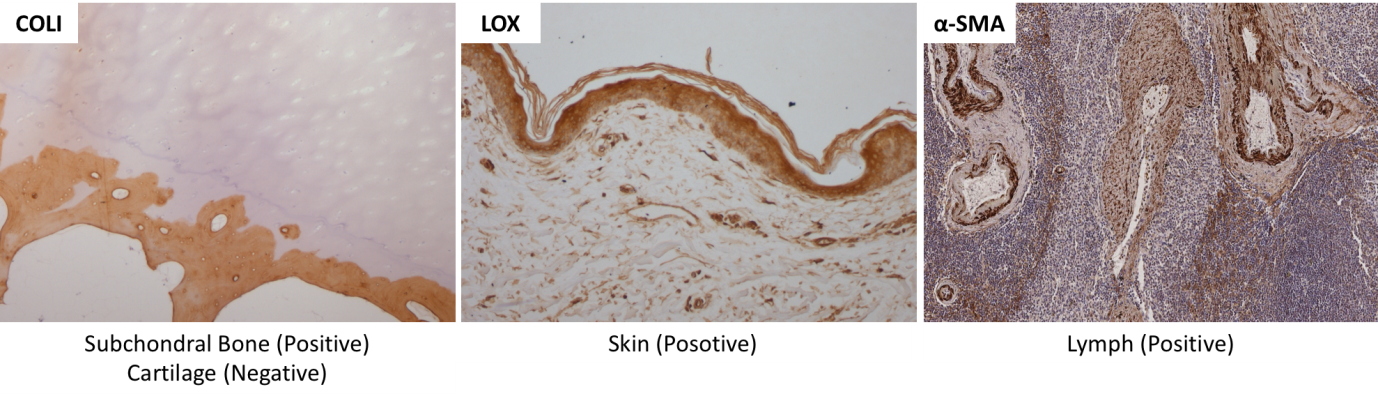

Supplement: Supplementary file 1 — Additional file 1: Figure S1. Positive/negative control for IHC of Collagen I (COLI), alpha-smooth muscle actin (α-SMA) and Lysyl Oxidase (LOX). [file 40001_2018_322_MOESM1_ESM.docx]
